# Molecular Insights into Phage–Hydrogel Polymer Interactions Through Docking, Molecular Dynamics, and Machine Learning

**DOI:** 10.3390/polym18080906

**Published:** 2026-04-08

**Authors:** Roba M. S. Attar, Mohammed A. Imam

**Affiliations:** 1 Department of Biological Sciences, College of Science, University of Jeddah, Jeddah 21959, Saudi Arabia; 2 Department of Medical Microbiology and Parasitology, Faculty of Medicine at Al-Qunfudah, Umm Al-Qura University, Al-Qunfudah 21961, Saudi Arabia; maimam@uqu.edu.sa

**Keywords:** bacteriophage delivery, hydrogels, molecular docking, MD simulations, machine learning, controlled release

## Abstract

An efficient bacteriophage delivery system needs to be developed to overcome the challenges associated with phage instability, rapid diffusion, and loss of infectivity at the infection site. Hydrogels have been found to be potential carriers. Hydrogels have emerged as promising carriers due to their biocompatibility, tunable physicochemical properties and capacity for controlled release. However, the molecular factors that regulate phage–hydrogel interactions remain poorly understood. In this study, we employed an in silico framework combining molecular docking, molecular dynamics (MD) simulations, MM/PBSA binding energy calculations, machine learning-based adhesion prediction, and diffusion modeling to explore phage–hydrogel interactions at the molecular level. Surface-exposed bacteriophage proteins, such as capsid and tail proteins, were evaluated against eight different hydrogel polymers. Binding site analysis revealed the presence of multiple solvent-accessible pockets that can interact with the polymer. Docking studies showed favorable and stable interactions, with hyaluronic acid showing strong binding affinity to multiple phage proteins (−5.5 to −5.7 kcal/mol) and GelMA showing high affinity to the capsid gp10 protein (−5.6 kcal/mol). The integrity of the structural complexes was further confirmed by 100 ns MD simulations, stable RMSD and RMSF trajectories, compact structural conformations, and favorable MM/PBSA binding energies. Machine learning classification successfully differentiated high- and low-adhesion systems and identified hydrogen bonding and electrostatic interactions as key determinants of sustained yet reversible phage retention. Collectively, our findings suggest that the hydrogels enriched with charged and polar functional groups can facilitate stable but non-destructive phage binding, enabling controlled and sustained release. This study provides mechanistic insights into rational hydrogel design for phage delivery systems and highlights the potential of high-throughput computational strategies to accelerate the development of optimized phage therapeutics.

## 1. Introduction

Infections due to antibiotic-resistant bacteria are predicted to cause tens of millions of deaths by 2050, thus putting emphasis on the need to develop alternative antimicrobial strategies [1]. The developing and spreading problem of multidrug-resistant organisms has had profound influences on the use of conventional antibiotics, especially in chronic and wound infections, where the formation of biofilms by bacteria and the prolonged colonization of bacterial pathogens have led to the failure of the healing process and unfavorable outcomes [2]. This emerging crisis, therefore, underscores the urgency in the search for alternative modalities that are considered to be potent in the selective, yet less toxic, targeting of resistant bacteria.

Bacteriophages, or simply phages, are viral entities that specifically kill or infect bacterial cells, and this has been identified as a new strategy in antimicrobial therapy. Unlike most antimicrobial agents, bacteriophages have specificity in action that also ensures their replication in infection sites, effectively killing the targeted bacterium without harming the host microbiota or human cells [3]. In addition, phages have inherent properties such as host specificity, ability to target and destroy bacterial biofilms using phage-encoded depolymerases, and good safety profiles. However, the successful application of phage therapy has been limited by challenges associated with phage stability and infectivity retention, as well as phage delivery and controlled release at the infection site. Therefore, the development of innovative phage delivery systems that can protect phages and ensure their sustained therapeutic activity at the infection site is essential for fully realizing the therapeutic potential of phage-based antimicrobial agents [4].

Adsorption is the initial step in phage infection. It is when bacteriophages specifically recognize and adhere to receptors on the surface of bacteria. This is mostly aided by tail-associated structural proteins in bacteriophages, such as tail fibers, tailspikes and tail baseplate receptor-binding proteins. These structures are a continuation of the distal tail area, resulting in host specificity, receptor recognition and permanent bacterial cell attachment [5,6].

**Figure 1:**
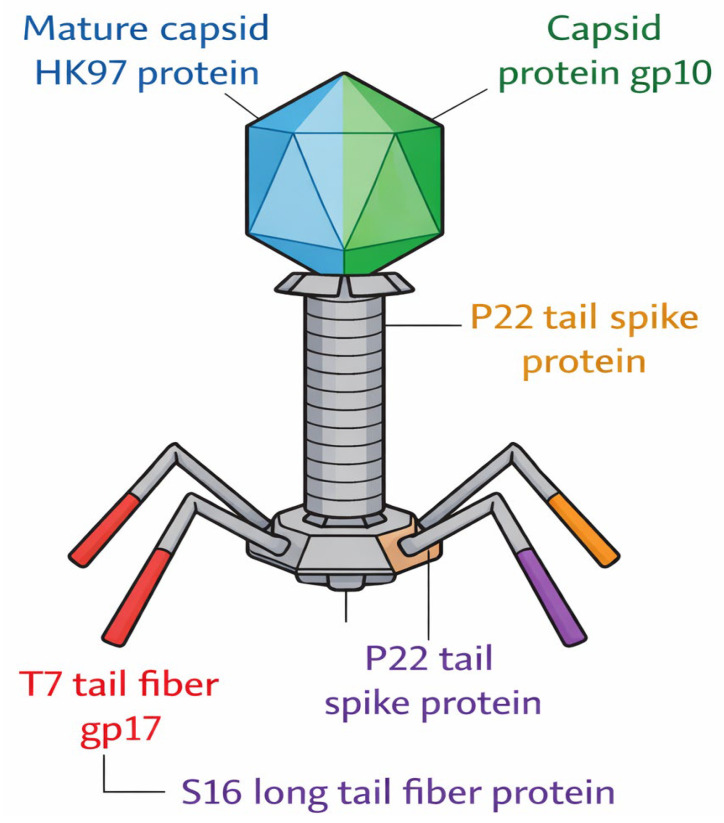
Schematic representation of a bacteriophage illustrating structural components involved in host detection and attachment.

Previous studies have shown that the genes responsible for these adsorption determinants are often located within the structural or morphogenetic components of the phage genome and are expressed at the late stage of infection [7]. Despite the variability in genomic organizations among phage families (Myoviridae, Siphoviridae and Podoviridae), adsorption-related genes are not evenly dispersed throughout the genome; rather, they are frequently concentrated inside the tail morphogenesis gene cassette [8]. A schematic representation of the phage adhesion is shown in Figure 1.

However, in practical therapy, free phage preparations are usually hampered by their instability at physiological temperature, fast diffusion away from the site of infection, and inactivation during administration, especially in cases of wound and implant infections [9]. Hydrogels have emerged as one of the most suitable delivery systems used to overcome these issues and ensure effective phage therapy [10]. Representative natural and synthetic hydrogels such as alginate, hyaluronic acid, carrageenan, chitosan, polyethylene glycol (PEG) and polyvinyl alcohol (PVA) were chosen for this study due to their widespread use in biomedical delivery systems and distinct physicochemical properties. These polymers differ in charge distribution, functional groups and hydrophilicity, allowing for a comparative analysis of how polymer chemistry affects phage adhesion and interaction behavior. These biocompatible polymeric materials are described as cross-linked networks that are non-toxic and highly hydrophilic, allowing them to absorb large amounts of water and create a protective, biocompatible microenvironment for biological agents [11]. Due to their high water content, hydrogels very closely resemble the structural and physicochemical properties of natural tissues, creating favorable conditions for the stabilization and encapsulation of proteins, living cells, and other biomolecules [12]. In addition, hydrogels possess predictable physicochemical properties and biodegradability, allowing for the precise control of release kinetics and their extension to the biomolecular delivery systems. In combination, these features underscore the highly promising nature of hydrogels as a system for the effective and selective delivery of bacteriophages [13].

Despite the experimental evidence of the possibility of the integration of bacteriophages into hydrogel matrices for the purpose of achieving antibacterial goals, the basic processes involved in the molecular interaction of phages and hydrogels are not well understood. Hydrogel-based antibacterial materials have gained considerable attention due to their ability to provide localized and sustained antimicrobial activity while maintaining a moist environment conducive to wound healing. Recent research has shown that polymeric hydrogels can serve as effective carriers for antibacterial medicines, allowing for regulated release and increased therapeutic efficacy. For example, improved polysaccharide-based hydrogels have been reported to display good antibacterial capabilities through increased polymer–microbe interactions and customizable physicochemical features that promote prolonged antimicrobial administration [14]. Similarly, recent advances in functional biomaterials have demonstrated the ability of designed polymeric networks to increase antibacterial effectiveness by improving structural stability, biocompatibility, and regulated drug or bioactive-chemical release patterns [15]. These studies highlight the expanding importance of hydrogel-based platforms in preventing bacterial infections, notably in biomedical applications including wound healing and implant-associated illnesses. The previous studies have mainly focused on the macroscopic observations of the viability, release rate, and antibacterial properties of polymeric hydrogel systems of PEG, alginate, and HPMC without determining the actual mechanisms of polymer–phage binding at the molecular level [16]. Research conducted on the impact of hydrogel elasticity, cross-link density, and mesh size on phage stability and release has demonstrated significant effects, yet not much information could be obtained concerning the phage binding residues, intermolecular forces, and molecular interactions, particularly those concerning the immobilization of the phages in the hydrogel networks [17]. The phage virus interactions are usually time-consuming, resource-consuming, and structure-limited, particularly because of the complex and heterogeneous nature of the hydrogel systems [18].

In silico approaches to the study of phage–hydrogel interactions have been identified as a potentially very useful tool to study the interaction between such a delivery system and the immune system at a molecular level, bypassing the challenges in gaining this knowledge using conventional approaches to wound healing. Molecular docking is a structure-based in silico tool for the efficient identification of optimal binding sites for the interaction between proteins derived from the bacteriophage capsid or tail fiber proteins and the polymeric chains of the hydrogels, in addition to a quantitative analysis of the binding affinities between these structural moieties, the predominance of electrostatic interactions and hydrogen bonding, and a number of other intermolecular interactions between these moieties [19]. Molecular dynamics simulations enable us to progress beyond the predictions related to the stability and flexibility of such a complex in solution in a physiological state representative of a wound healing scenario related to ionic strength, pH, and temperature conditions [20]. Beyond atomistic simulations, machine learning (ML) models can be explored for efficient integration, particularly towards incorporating multi-scale physicochemical properties and simulation-based features, with respect to prediction methodologies for phage adhesion efficiency, stability, and retention onto different hydrogel material scaffolds [21].

Concurrently, diffusion- and release-related modeling aids in the quantified study of macroscopic processes of releasing phages as a further extension of molecular-level interactions, paving the path to rational prediction of mobility, immobilization, and releasing capabilities of phages within hydrogels designed to mimic hydrated wound tissue and implant surfaces [22]. Despite increasing experimental studies on phage-loaded hydrogel systems, the majority of existing research focuses on assessing antibacterial activity, phage survivability and release kinetics at the macroscopic level. Understanding the molecular mechanisms driving phage–polymer adhesion and stability within hydrogel matrices has received relatively limited attention. In particular, the discovery of individual binding residues, intermolecular interactions and structural factors relevant for phage immobilization remains largely unexplored. Furthermore, previous investigations have rarely combined molecular-level simulations with predictive computational modeling tools to systematically assess phage–hydrogel interactions. To address these constraints, this study proposes a complete multi-scale in silico framework that incorporates molecular docking, molecular dynamics simulation, machine learning-based adhesion prediction and diffusion modeling. This integrated approach provides mechanistic insights into the interaction of bacteriophage structural proteins with various hydrogel polymers, allowing for predictions of phage adhesion efficiency, stability and release behavior. Unlike previous studies, which rely primarily on experimental observations, the current study proposes a computational strategy for linking molecular interactions to macroscopic diffusion behavior, thereby contributing to the rational design of hydrogel-based phage delivery systems for wound and implant-associated infections.

## 2. Materials and Methods

### 2.1. Phage and Hydrogel Retrieval

The three-dimensional structures of bacteriophages’ surface-exposed proteins were chosen to represent the important molecular interfaces that are involved in the phage–hydrogel interactions. The capsid proteins, tail fiber proteins, and receptor-binding proteins were chosen based on a thorough literature survey of bacteriophages that have been previously characterized for antibacterial and biomedical applications. The structurally resolved protein models with high integrity were obtained from the Protein Data Bank (PDB) (https://www.rcsb.org/, accessed on 28 October 2025) [23].

To represent the chemical properties of hydrogel matrices at the molecular level, the representative monomeric building blocks of commonly used biomedical hydrogel polymers were obtained from the PubChem database (https://pubchem.ncbi.nlm.nih.gov/, accessed on 30 October 2025) [24]. These monomers are the basic repeating units of polymeric networks such as chitosan, alginate, polyethylene glycol (PEG), and other biocompatible hydrogels. The three-dimensional structures of the chosen monomers were downloaded in Structure Data File (SDF) format for further computational analysis. Since hydrogel polymers are vast cross-linked networks, molecular docking and molecular dynamics simulations were performed on representative monomeric units from each hydrogel polymer (alginate, hyaluronic acid, carrageenan, chitosan, polyethylene glycol and polyvinyl alcohol). The molecular structures of these polymer units were derived from publicly available chemical databases and optimized prior to docking analysis.

The choice of phage surface proteins and monomeric units of the hydrogel was made based on their biological relevance. This approach allows for a simplified but biologically relevant modeling of the phage–hydrogel systems, which can be used for molecular docking, interaction analysis, and molecular dynamics simulations to understand the mechanisms of phage immobilization and release in hydrogel matrices.

### 2.2. Structure Preparation and Molecular Modeling

All phage protein structures and units of hydrogel monomers were optimized and prepared before the analyses of molecular interactions. The software UCSF ChimeraX (Version 1.11.1, accessed on 29 October 2025) [25] was used for protein structure preparation. It offers well-tested biomolecular preprocessing tools and energy minimization algorithms.

In the case of bacteriophage surface proteins, only the surface-exposed chains that are of interest for molecular interactions were kept, and the unnecessary chains were eliminated. In cases where multiple models were present in the structure, a representative model was chosen to eliminate redundancy. The protein structures were then cleaned by eliminating crystallographic details such as solvent molecules, ions, and ligands that were co-crystallized with the proteins and could affect the accuracy of molecular docking and simulations.

Hydrogen atoms were added to all protein structures to model the protonation states that are not resolved in X-ray crystallographic structures, thus allowing for the correct calculation of electrostatic interactions. Atomic partial charges were assigned and energy minimization was done using the Dock Prep module of ChimeraX to eliminate steric overlaps and optimize local geometry. Protein minimization was done using the AMBER ff14SB force field with default settings, which typically involves around 200 steps of minimization; this was done to ensure that energetically stable structures were obtained without disturbing the native protein fold.

Components of the hydrogel were modeled separately at the molecular level. Representative monomeric subunits of hydrogel polymers were extracted from the PubChem database in three-dimensional geometry and geometry-optimized. For hydrogel systems that are based on more complex repeating units, such as carrageenan, molecular modeling was done using Avogadro software (https://avogadro.cc/, version 1.2, accessed on 30 October 2025) [26]. Representative units were built manually, paying attention to stereochemistry and glycosidic bonds. These structures were further optimized to ensure correct bond geometry and conformational stability for further interaction studies. All proteins and hydrogels were transformed into appropriate file formats and validated before further analysis.

### 2.3. Binding Site Prediction

To facilitate biologically meaningful and computationally feasible molecular docking, potential interaction sites on the surface proteins of the phage were determined before the analysis of interaction. The prediction of the binding site was carried out using the CASTpFOLD server (https://cfold.bme.uic.edu/castpfold/, accessed on 1 November 2025) [27], which predicts surface-accessible pockets and cavities on the basis of protein topology and geometric properties, such as the volume and surface area of the pocket.

The identification of the potential interaction site is an essential prerequisite for protein–ligand docking studies, especially in the case of large biomolecular systems like bacteriophage surface proteins, which have large solvent-accessible surfaces [28]. Blind docking on the whole protein surface can lead to non-specific or energetically unfavorable binding conformations and is computationally expensive. Hence, the site-directed docking approach was employed to study the biologically relevant binding sites [29].

On the basis of CASTpFOLD analysis, the most dominant pockets in terms of volume, dimension, and accessibility have been identified as pivotal for interaction. These have been used for the generation of docking grids, which have been utilized in site-directed docking approaches that permitted the selective placement of hydrogel monomeric units in areas of high probability of involvement in interaction with the phage.

By constraining the space in which the ligand docking search was conducted to biologically plausible loci, the “site-directed docking” approach was able to facilitate the accurate prediction of ligand orientation and intermolecular interactions.

### 2.4. Molecular Docking

Molecular docking was used in order to investigate the interaction between the hydrogel monomeric units and the surface proteins of the bacteriophages. Molecular docking was performed using the PyRx software program (https://pyrx.sourceforge.io/ version 0.8, accessed on 5 November 2025). This software program uses AutoDock Vina (v1.2.5) to facilitate the process of docking [30].

The prepared phage surface protein structure was used as the receptor, and the optimized hydrogel monomeric units were used as ligands. Site-directed docking was performed, where the docking grids were defined according to the predicted interaction pockets identified from the binding site analysis. This enabled the specific placement of the ligand in biologically relevant areas, thus minimizing the chances of non-specific binding.

Various binding poses were created, and they were ranked based on the predicted binding affinity values for the specific protein–ligand complexes. The docked molecules were evaluated with reference to the values of the calculated binding energy and the RMSD values obtained during the prediction. The docking studies that met the specific criteria for selecting the docked molecules were chosen for evaluation. The selection was based on the ability of hydrogel monomers to form stable binding poses in the predicted interaction regions, which reflected the potential of phage–hydrogel interactions.

To better understand the docking scores, the interaction analysis of the docked complexes was carried out using different molecular visualization software. Two-dimensional interaction maps showing hydrogen bonding, hydrophobic interactions, electrostatic interactions, and π-interactions were created using Discovery Studio Visualizer 2025 (https://www.3ds.com/products/biovia/discovery-studio, accessed on 12 November 2025) [31]. Three-dimensional structural visualization and orientation analysis were done using PyMOL (https://www.pymol.org/ version 3.1, accessed on 15 November 2025) [32].

### 2.5. Molecular Dynamics (MD) Simulations

Molecular dynamics simulations were carried out using the Desmond module of the Schrödinger suite (Version 2025-4, accessed on 10 December 2025) to analyze the stability and dynamics of the chosen protein–ligand complexes [33]. Preparation of the protein–ligand complexes was done before the simulation with the aid of the Protein Preparation Wizard. The complexes were parameterized by the use of the OPLS-2005 force field, widely used in biomolecular simulations [34].

Each of the prepared complexes was solvated in an orthorhombic simulation box with the TIP3P water model, while maintaining a minimum distance of 10 Å between the solute and the boundary of the box. Sodium (Na^+^) and chloride (Cl^−^) ions were added as needed to neutralize the systems and achieve physiological ionic strength. Energy minimization, to remove steric clashes and unfavorable interactions, was followed by a multistep equilibration protocol [35].

The MD simulation was carried out for 100 ns in NPT conditions at 310 K and 1 atm. Regularly, trajectory frames were saved in order to perform an analysis of the results obtained in the simulation, which included RMSD, RMSF, and MM-GBSA algorithms for the computation of the free energy of binding [36].

### 2.6. Machine Learning-Based Prediction of Adhesion Efficiency

To improve studies aimed at assessing and characterizing the efficiency of adhesion between phage and hydrogel, a binary classification approach via ML was also developed. With this approach, a range of descriptions of molecular interactions was integrated for a better and more cohesive understanding, complementing the information obtained with docking and MD simulations.

The molecular docking and MD simulation analysis also produced many other descriptors for the stability and binding of the phage with the hydrogel. The descriptors obtained include the average root mean square deviation (RMSD), average hydrogen bonds, average binding free energy for MM-PBSA/MM-GBSA, average solvent surface area, and adhesion labels, which describe the outcomes of the binding event. The obtained descriptors have relevance for providing information regarding many of the factors governing the adhesion efficiency, as there is concern for structural stability, hydrogen bonds, binding energy, and surface areas.

In this study, logistic regression was used as the classifier since it is easier to interpret, especially in handling datasets of small to medium size, as is often the case in studies involving biomolecular simulation [37]. The classifier was trained to distinguish between the systems with efficient adhesion and the systems with inefficient adhesion based on the feature set that was identified.

In order to confirm the effectiveness of the model and avoid cases of overfitting, the leave-one-out cross-validation (LOOCV) method was employed. This method, as suggested by [38], allows all of the available data to be utilized as much as possible. This method was chosen due to the nature of the dataset, which was small in quantity and extracted computationally. In the LOOCV approach, all of the dataset was utilized in turns, while the remaining portion was utilized for training.

In a nutshell, the ML classification model enabled a systematic prediction of adhesion efficiency by incorporating structural, energetic, and surface-related features. This approach gave an additional validation on the stability of phage–hydrogel interactions and provided a way to identify the best hydrogel candidates for efficient phage immobilization.

### 2.7. Diffusion and Release Modeling

The diffusion and release properties of bacteriophages contained in different kinds of hydrogel matrices were simulated with the Higuchi diffusion model, which is actually a diffusion-controlled release model for various kinds of polymers [39]. This simulation was done according to the macroscopic properties based on the interaction strength between the phages and the hydrogel.

To perform the diffusion and release modeling, the scripts were created using the programming language Python, which was useful in incorporating parameters of molecular interactions acquired from molecular docking, molecular dynamics simulations, and machine learning algorithms concerning adhesion efficiency. For the estimation of the diffusion coefficient (D_{eff}) of the individual phage–hydrogel complexes, the molecular adhesion strength was considered, including the binding free energy, the entire stability of interactions, hydrogen bond formation, and machine learning calculations of adhesion efficiency.

The Higuchi model was applied according to the following equation:

$$Q=k_{H}\sqrt{t}$$

where *Q* is the amount of phage released per unit surface area, *t* is time, and *k_H_* is the Higuchi release constant, which is proportional to the diffusing velocity of the phage within the hydrogel system. In the present work, *k_H_* was varied according to the estimated D_eff, which links adhesion properties to release kinetic phenomena.

The basic assumption of this approach to modeling is as follows: increased adhesion force between a phage and a hydrogel is equivalent to decreased molecule mobility and increased rates of release, and, conversely, a low adhesion force is equivalent to increased molecule mobility and fast rates of release [40]. The model makes use of diffusivity obtained from adhesion forces and calculated by means of a Higuchi equation, thereby accounting for altered rates of phage adhesion owing to changes in adhesion force rather than material properties themselves.

The Python-based diffusion release modeling concept has helped in the comparative modeling of phage release kinetics in different hydrogel matrices, and it has established a fundamental connection between release kinetics and molecular-level encounters.

## 3. Results

### 3.1. Structural Characterization of Phage Surface Proteins and Hydrogel Components

The three-dimensional structures of bacteriophage surface proteins important in host recognition, adsorption, and capsid stability were extracted from the Protein Data Bank (PDB) and processed for additional computer study. Prior to interaction analysis, all protein structures were improved by removing non-standard residues and superfluous atoms, then adding polar hydrogen atoms with UCSF ChimeraX. These proteins include the long tail fiber protein of bacteriophage S16 (PDB ID: 6F45), the tailspike protein of bacteriophage P22 (PDB ID: 1TSP), and the carboxy-terminal region of the bacteriophage T7 tail fiber protein gp17 (PDB ID: 4A0T). These proteins have a direct role in bacterial host attachment and the initial adsorption stage, making them biologically appropriate models for researching phage adhesion behavior on hydrogel.

Aside from the proteins found in bacteriophages’ tails, the structure of the mature capsid was used to predict the surface proteins of phages involved in structural integrity and, presumably, non-specific surface contacts. The mature capsid structure (PDB ID: 1OHG) and mature capsid protein gp10 (PDB ID: 6OMC) were utilized to investigate interaction patterns on a larger surface area of the phage that was not involved in receptor-binding.

The structural analysis revealed that the surface-exposed residues and functional domains of the retrieved phage proteins were well-resolved, making them amenable for molecular docking and interaction analysis.

The representative hydrogel polymer building blocks of chitosan, polyethylene glycol (PEG), polyvinyl alcohol (PVA), and others (Table 1) were retrieved from the PubChem database and energy-minimized before interaction analysis. These polymers were chosen for their well-established biocompatibility, adjustable physicochemical properties, and extensive use in biomedical delivery systems. The molecular visualization analysis confirmed the presence of functional hydroxyl and amine groups that are capable of hydrogen bonding and electrostatic interactions with the phage surface residues.

### 3.2. Binding Site Identification on Bacteriophage Surface Proteins

The Computed Atlas of Surface Topography of Proteins (CASTpFOLD) server was used to screen for possible binding pockets and active sites on the target bacteriophage surface proteins. This was done to assess the solvent-accessible surface area and volume that could be occupied by the hydrogel polymer components in the docking simulation.

For the tailspike protein of bacteriophage P22 (PDB ID: 1TSP), the CASTpFOLD analysis showed a large binding pocket with a solvent-accessible surface area of 1045.522 Å^2^ and a volume of 2158.931 Å^3^, which is highly accessible at the binding interface. However, for the mature capsid (PDB ID: 1OHG), a smaller binding pocket was observed, with a surface area of 120.482 Å^2^ and a volume of 58.714 Å^3^, which is less accessible due to the limited surface area of the cavity.

Likewise, the carboxy-terminal domain of the bacteriophage T7 tail fiber protein gp17 (PDB ID: 4A0T) had a binding pocket with a surface area of 64.146 Å^2^ and a volume of 24.558 Å^3^, which was in line with a localized receptor-binding site. On the other hand, the long tail fiber protein of bacteriophage S16 (PDB ID: 6F45) had a binding pocket with a surface area of 562.116 Å^2^ and a volume of 1247.163 Å^3^, which was indicative of a higher potential for polymer-mediated interactions. The mature capsid protein gp10 (PDB ID: 6OMC) had a binding pocket with a surface area of 349.010 Å^2^ and a volume of 158.642 Å^3^, which was indicative of moderate accessibility compared to the other proteins studied. The identified binding pockets were then chosen as docking sites for the study of phage–hydrogel interactions.

### 3.3. Docking Analysis of Phage–Hydrogel Interactions

Molecular docking and virtual screening experiments were carried out to assess the interaction potential of bacteriophage surface proteins with the hydrogel polymer components. Eight model hydrogel building blocks (chitosan, alginate, hyaluronic acid, carrageenan, polyethylene glycol (PEG), polyvinyl alcohol (PVA), gelatin methacryoyl (GelMA), and 2-Hydroxyethyl methacrylate (HEMA)) were docked into the predicted binding sites of each phage protein (Table 2).

Docking experiments were carried out using the PyRx software tool using a multi-ligand docking strategy. Binding affinity scores, root mean square deviation (RMSD) values, hydrogen bond counts, and interacting amino acid residues were evaluated for each phage–hydrogel system to assess the interaction strength and stability.

#### 3.3.1. Docking of Hydrogel Polymers with Mature Capsid Structure (PDB ID: 1OHG)

**Figure 2:**
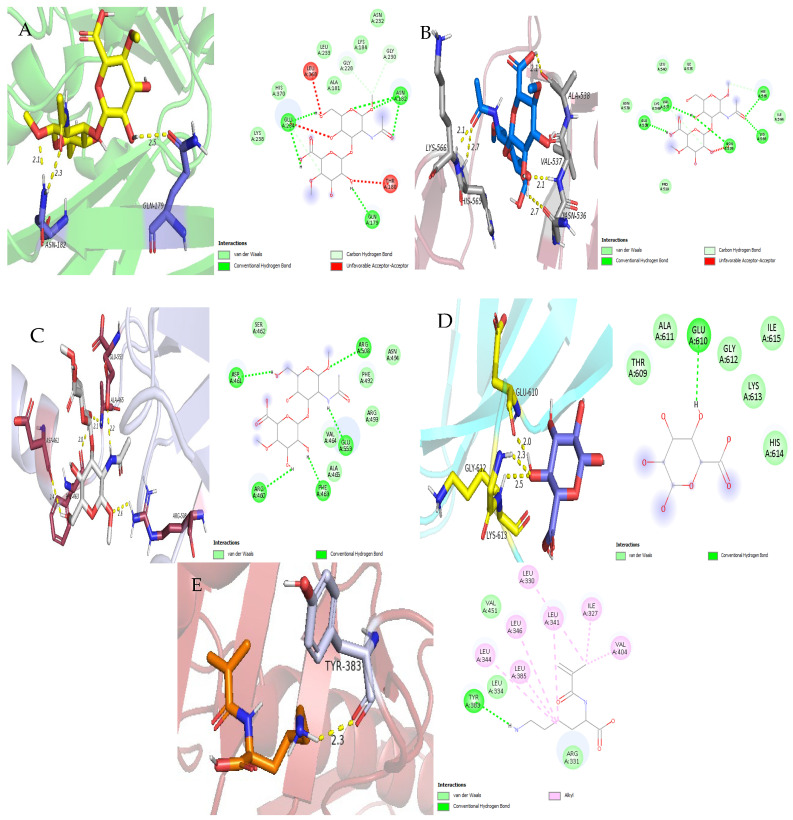
3D and 2D interactions between hydrogel monomers and phage surface proteins. (**A**) Hyaluronic acid with mature capsid (PDB ID: 1OHG); key hydrogen bonds are shown as dashed green lines; electrostatic interactions are highlighted in red; interacting residues labeled. (**B**) Hyaluronic acid with P22 tailspike (1TSP) protein; hydrogen bonds and π- interactions are indicated. (**C**) Hyaluronic acid with T7 tail fiber gp17 (4A0T). (**D**) Alginate with S16 long tail fiber (6F45). (**E**) GelMA with capsid gp10 (6OMC). 2D maps show hydrogen bonding (green), hydrophobic (pink) and electrostatic (red) interactions: Only residues within 5 Å of the ligands are shown.

The molecular docking experiment showed that there were stable interactions between the mature capsid protein structure (PDB ID: 1OHG) and the hydrogel polymers. The highest binding affinity was shown by hyaluronic acid with a binding affinity of −5.5 kcal/mol and an RMSD of 1.26 Å, which is an indication of a stable binding conformation. The interaction analysis showed that the hyaluronic acid molecule formed hydrogen bonds with the side chains of GLU A: 234, ASN A: 182, and GLN A: 179 (Figure 2A).

Alginate also showed a high binding affinity with the mature capsid protein structure, with a binding affinity of −5.1 kcal/mol and an RMSD of 0.69 Å. The hydrogen bond interaction was observed between the alginate molecule and the side chains of ASN A: 182 and ASN A: 232.

In general, the results of the docking analysis showed that the binding affinities of hyaluronic acid and alginate were higher towards the mature form of the capsid structure than the other hydrogel polymers. The binding affinities of chitosan (−4.9 kcal/mol), carrageenan (−4.8 kcal/mol), and GelMA (−4.6 kcal/mol) were moderate.

#### 3.3.2. Docking of Hydrogel Polymers with P22 Tailspike Protein (PDB ID: 1TSP)

The molecular docking study showed positive binding interactions between the hydrogel polymers and the P22 tailspike protein. Hyaluronic acid showed the highest binding affinity, with a docking score of −5.7 kcal/mol and RMSD of 0.49 Å, which showed high binding specificity and a stable binding conformation. Interaction analysis showed that hyaluronic acid interacted with the side chains of ALA A: 538, VAL A: 537, ASN A: 536, and LYS A: 566 through multiple hydrogen bonds, which showed strong and specific binding interactions in the predicted binding site (Figure 2B).

Alginate also showed a positive binding profile with the P22 tailspike protein, with a binding affinity of −4.7 kcal/mol and RMSD of 1.06 Å. Hydrogen bond interactions were observed with VAL A: 537 and ILE A: 535.

Other hydrogel polymers had moderate binding affinities. Carrageenan had a binding affinity of −4.7 kcal/mol with an RMSD of 1.96 Å, while Chitosan and GelMA had binding affinities of −4.3 kcal/mol with RMSDs of 0.49 Å and 1.25 Å, respectively. In general, the docking study revealed that hyaluronic acid and alginate have higher binding affinities to the P22 tailspike protein than other hydrogel polymers.

#### 3.3.3. Docking of Hydrogel Polymers with T7 Tail Fiber gp17 (PDB ID: 4A0T)

Molecular docking studies of hydrogel polymers with the carboxy-terminal domain of the bacteriophage T7 tail fiber protein gp17 revealed stable and consistent interaction patterns among the tested hydrogels. Among the tested polymers, hyaluronic acid showed the highest binding affinity, with a docking score of −5.7 kcal/mol and an RMSD value of 1.70 Å, suggesting a favorable and stable binding conformation within the predicted interaction site. Hydrogen bond analysis showed that hyaluronic acid formed interactions with the side chains of ASP A: 461, ARG A: 460, PHE A: 463, GLU A: 553, and ARG A: 508, which helped in the stabilization of the protein–polymer complex (Figure 2C).

Carrageenan also demonstrated specific binding to the T7 tail fiber gp17 protein, with a binding affinity of −4.8 kcal/mol and an RMSD of 0.83 Å, indicating proper orientation within the binding pocket.

The other hydrogel polymers demonstrated moderate binding affinity. Alginate had a binding affinity of −4.7 kcal/mol, while chitosan and GelMA had binding affinities of −4.4 kcal/mol and −4.9 kcal/mol, with RMSD values of 0.45 Å and 1.63 Å, respectively. However, the docking study revealed that hyaluronic acid and carrageenan had higher binding affinity to the T7 tail fiber gp17 protein than the other hydrogel polymers tested.

#### 3.3.4. Docking of Hydrogel Polymers with S16 Long Tail Fiber Protein (PDB ID: 6F45)

Molecular docking studies of hydrogel polymer with the long tail fiber protein of bacteriophage S16 revealed moderate binding affinities among the tested hydrogels. Hyaluronic acid showed the highest binding affinity, with a docking energy of −4.7 kcal/mol and an RMSD value of 0.61 Å, which revealed a stable binding conformation. The interaction studies showed that hyaluronic acid made interactions with the side chains of HIS A: 614, GLY A: 612, and THR A: 609 residues, which helped in the stabilization of the complex at the predicted binding pocket.

Alginate also had a favorable binding affinity with the S16 long tail fiber protein, with a binding affinity of −3.7 kcal/mol and an RMSD of 0.48 Å. A hydrogen bond interaction with GLU A: 610 was also seen, indicating the presence of localized electrostatic interactions in the complex (Figure 2D).

The other hydrogel polymers had relatively lower binding affinities. Carrageenan had a binding affinity of −3.7 kcal/mol with an RMSD of 1.13 Å, while Chitosan and GelMA had binding affinities of −3.4 kcal/mol and −3.3 kcal/mol with RMSD values of 0.31 Å and 1.15 Å, respectively. The comparative docking studies revealed that hyaluronic acid had the most favorable interaction with the S16 long tail fiber protein, followed by alginate, while the other hydrogels had moderate to weak binding properties.

#### 3.3.5. Docking of Hydrogel Polymers with Capsid Protein gp10 (PDB ID: 6OMC)

Molecular docking studies of hydrogel polymers with the capsid protein gp10 of bacteriophage (PDB ID: 6OMC) showed variations in the binding affinity of the different hydrogels tested. Gelatin methacryloyl (GelMA) showed the highest binding affinity to the gp10 protein, with a docking score of −5.6 kcal/mol and an RMSD of 1.75 Å, suggesting a stable binding conformation. The interaction study revealed that GelMA had a hydrogen bond interaction with the side chain of THR A: 383, which helped in the stabilization of the protein–polymer complex (Figure 2E).

Hyaluronic acid showed a high binding affinity to the capsid protein gp10, with a binding affinity of −4.7 kcal/mol and an RMSD of 1.62 Å. Hydrogen bond interactions were observed with the residues ARG A: 336, ARG A: 332, LYS A: 333, and LEU A: 334, suggesting that hydrogen bonding was responsible for the stabilization of the protein–ligand complex in the predicted binding site.

The other hydrogel polymers had relatively moderate binding affinities. Chitosan and polyvinyl alcohol (PVA) had binding affinities of −4.6 kcal/mol with RMSDs of 0.13 Å and 0.48 Å, respectively. Another polymer had a binding affinity of −4.3 kcal/mol with an RMSD of 0.71 Å. These data collectively suggest that GelMA and hyaluronic acid have higher binding affinities to the capsid protein gp10 than the other hydrogel polymers evaluated.

In general, the molecular docking study showed both similarities and differences in the binding affinities of hydrogel polymers to the bacteriophage surface proteins studied. For all five phage proteins, hyaluronic acid had the highest binding affinity, followed by alginate and GelMA. Chitosan, carrageenan, PEG, PVA, and HEMA, on the other hand, had relatively moderate binding affinities.

The improved binding affinity of hyaluronic acid was linked to relatively low RMSD values and the presence of multiple hydrogen bonds with exposed amino acid residues of tail fiber, tailspike, and capsid proteins. Such properties can be ascribed to the high density of hydroxyl and carboxyl functional groups in hyaluronic acid molecules, as well as the flexibility of the molecule, which allows for diverse hydrogen bonding interactions with polar and charged amino acid residues. Such properties may be responsible for its ability to bind in structure-independent binding modes to diverse phage proteins.

Based on these docking results, the chemical composition, the density of the functional groups, and the accessibility of the binding pockets are significant factors in the binding affinity of the phage–hydrogel interactions. Out of the tested polymers, the hyaluronic acid polymer exhibited the best mode of interaction for the screened phage surface proteins.

### 3.4. Molecular Dynamics Simulations Analysis

Molecular dynamics (MD) simulations were performed for some of the phage–hydrogel polymer complexes over a timescale of 100 ns to validate and interpret the results obtained by molecular docking experiments. The phage–polymer complexes selected were hyaluronic acid–mature capsid (1OHG); alginate–mature capsid (1OHG); hyaluronic acid–T7 tail fiber gp17 (4A0T); carrageenan–T7 tail fiber gp17 (4A0T); hyaluronic acid–S16 long tail fiber (6F45); alginate–S16 long tail fiber (6F45); GelMA–capsid protein gp10 (6OMC); and hyaluronic acid–capsid protein gp10 (6OMC). Calculations were done by using various parameters like MM/PBSA binding free energy, root mean square deviation, root mean square fluctuation radius, and solvent-accessible surface area.

To guarantee a clear presentation devoid of repetition, selected typical phage–hydrogel complexes were utilized for in-depth structural and dynamic visualization while quantitative molecular dynamics findings for all simulated systems are included in the accompanying tables.

#### 3.4.1. MM/PBSA Binding Free Energy Analysis

MM/PBSA binding free energy calculations showed that all complexes had favorable binding energies, thus verifying that the non-covalent interactions between the polymers and the phage proteins were stable throughout the simulation. Among the capsid protein complexes, the hyaluronic acid showed a stronger binding affinity than alginate. In this complex, the hyaluronic acid showed a binding affinity of −41.65 kcal/mol, whereas alginate showed a weaker binding affinity of −30.69 kcal/mol. With regard to the P22 tailspike protein complex, carrageenan showed the strongest binding energy among the polysaccharides, with a binding energy of −47.29 kcal/mol, followed closely by hyaluronic acid, showing a binding energy of −44.51 kcal/mol. In the T7 tail fiber protein complex, hyaluronic acid showed a stronger binding affinity than carrageenan. Specifically, in this complex, hyaluronic acid showed a strong binding affinity of −52.32 kcal/mol, whereas carrageenan showed merely half of that with a binding affinity of −35.39 kcal/mol. With regard to the S16 long tail fiber protein complex, alginate showed a stronger binding affinity than hyaluronic acid (Table 3).

#### 3.4.2. RMSD Analysis

**Figure 3:**
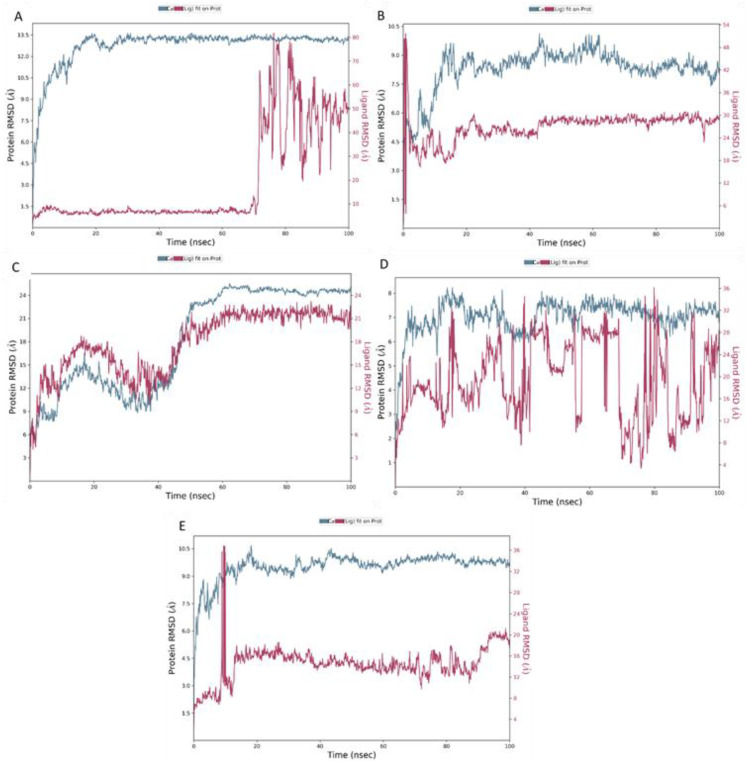
Protein backbone and polymer RMSD profiles from 100 ns molecular dynamics simulations of bacteriophage surface protein–hydrogel polymer complexes. (**A**) Hyaluronic acid–mature capsid (1OHG), (**B**) hyaluronic acid–P22 tailspike protein (1TSP), (**C**) hyaluronic acid–T7 tail fiber protein gp17 (4A0T), (**D**) alginate–long tail fiber protein of bacteriophage S16 (6F45) and (**E**) GelMA–capsid protein (6OMC). Protein RMSD (blue, left axis) reflects conformational stability of the phage proteins while polymer RMSD relative to the protein (purple, right axis) captures ligand flexibility and binding dynamics over the simulation timeframe.

All the protein–polymer complexes were found to have initial convergence and were found to be stable with little fluctuation throughout the 100 ns simulation, supporting the conclusion that the systems were at equilibrium and had structural integrity. The results indicate that the hydrogel polymers were able to form stable protein complexes with the surface proteins of the phages. Figure 3 displays the representative RMSD plots.

#### 3.4.3. RMSF Analysis

**Figure 4:**
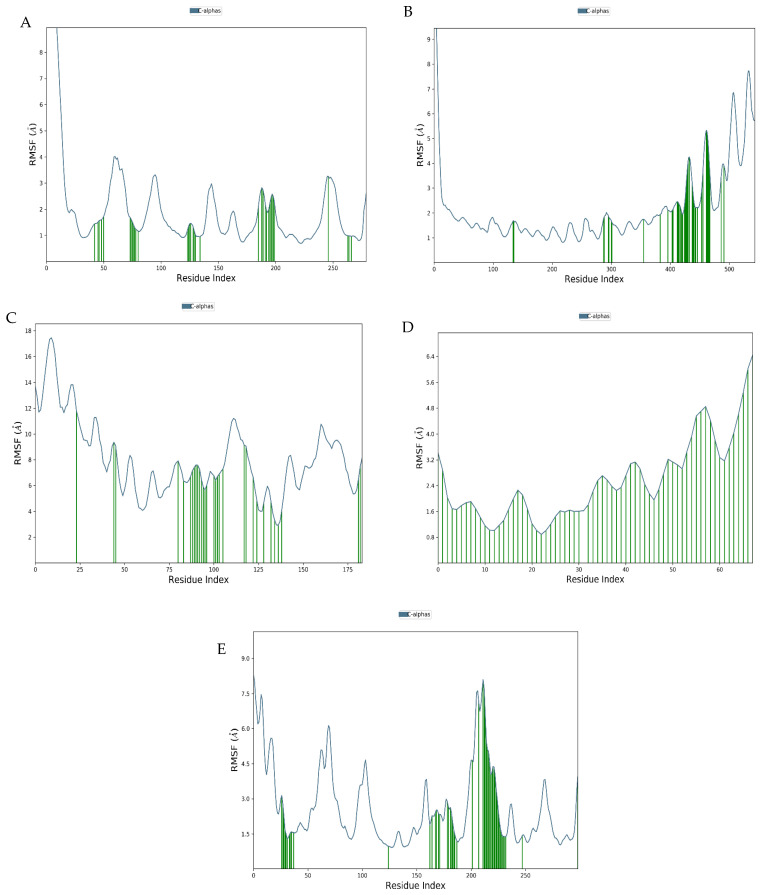
RMSF profiles of Cα atoms from 100 ns molecular dynamics simulations of bacteriophage surface protein–hydrogel complexes. (**A**) Hyaluronic acid–mature capsid (1OHG), (**B**) hyaluronic acid–P22 tailspike protein (1TSP), (**C**) hyaluronic acid–T7 tail fiber gp17 (4A0T), (**D**) alginate–long tail fiber protein of bacteriophage S16 (6F45) and (**E**) GelMA–capsid protein (6OMC). RMSF values are plotted as a function of residue index and reflect residue level flexibility of the phage proteins in the presence of bound hydrogels.

The residue level flexibility was also investigated through RMSF calculations. The results indicated the lack of significant residue level fluctuations for most of the residues, while enhanced flexibility was identified for the terminal and loop regions only, as expected for large protein molecules. The RMSF calculations carried out for the ligands also showed a lack of significantly fluctuating atoms, thereby ensuring a proper binding of the polymers (Figure 4).

#### 3.4.4. Radius of Gyration (Rg) Analysis

**Figure 5:**
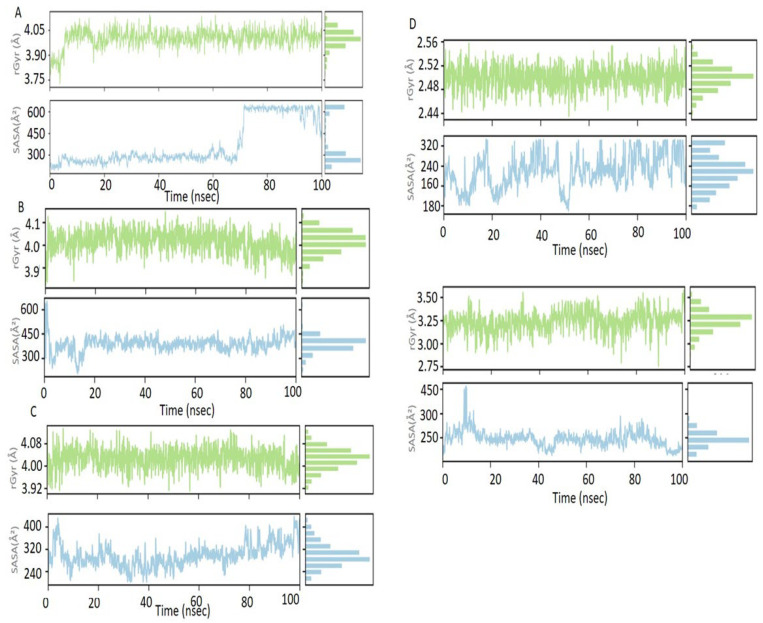
Radius of gyration (Rg) and solvent-accessible surface area (SASA) analysis of representative phage–hydrogel complexes during 100 ns molecular dynamics simulations.

During the analysis, the values of the radius of gyration indicated that all the complexes retained compact structural conformations during the simulation process. These slight changes in Rg values indicate that the protein folding was maintained, showing stability as the complexes optimized their molecular interactions. Figure 5 shows the temporal evolution of the radius of gyration (Rg) and solvent-accessible surface area (SASA) for several phage–hydrogel complexes.

#### 3.4.5. Solvent-Accessible Surface Area (SASA) Analysis

The deviation of the SASA values was very minimal over 100 ns for all the complexes, which suggests that the proteins were folded properly with the least exposure of the hydrophobic cores, thereby validating the structural stability of the protein–polymer complexes. According to the Rg trends, the SASA profiles fluctuated minimally during the simulation, indicating that the protein folding and polymer interaction were intact.

#### 3.4.6. Protein–Ligand Interaction Profiles

**Figure 6:**
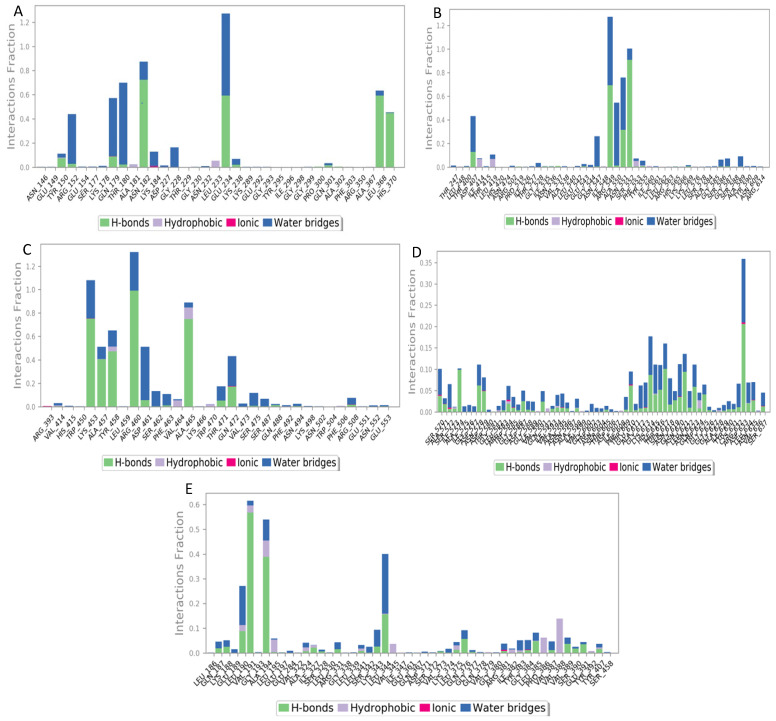
Residue-wise phage–hydrogel interaction profiles derived from 100 ns molecular dynamic simulations. Each bar represents the contribution of individual amino acid residues involved in binding interactions. Interaction types are categorized as hydrogen bonds, hydrophobic contacts, ionic interactions, water bridges and van der Waals interactions. Color coding corresponds to different interaction types as indicated in the legend.

The protein–ligand interaction profiles confirmed that the hydrogel polymers formed hydrogen bonding, electrostatic, and hydrophobic interactions with specific amino acid residues of the proteins from the bacteriophages. The interaction between these proteins and the hydrogel polymers ultimately stabilizes the complex, and these types of interaction are consistent with the positive binding energies calculated from the MM/PBSA method. Persistent connections detected across trajectories reveal critical residues involved in the dynamic stability of phage–hydrogel complexes (Figure 6).

### 3.5. Machine Learning-Based Prediction of Phage–Hydrogel Adhesion Efficiency

To evaluate whether descriptors of interaction obtained by means of MD simulations could be potentially used as predictors in adhesion efficiency between a phage and a hydrogel, a supervised ML method was proposed and implemented for ten different phage and hydrogel combinations. The information fed to create the ML model comprised the most significant structural, energetic, and interfacial data obtained from MD simulation interaction descriptors, such as root mean square deviation (RMSD), the number of hydrogen bonds, binding free energy (ΔG), and solvent-accessible surface area (SASA).

RMSD_avg and SASA_avg are average values derived over the last 20 ns of a 100 ns simulation. Hbond_avg represents the average number of hydrogen bonds established during the simulation. The MM/PBSA method was used to calculate ΔG_bind values, with negative values indicating more favorable binding. Adherence labels represent binary classification outcomes (1 = strong adhesion, 0 = weak adhesion) (Table 4).

A binary classification approach (high vs. low adhesion efficiency) was used with a logistic regression classifier, and the class labels were assigned using median-based thresholding of the MD simulation-derived interaction descriptors. Due to the small size of the dataset (*n* = 10), leave-one-out cross-validation (LOOCV) was used.

**Figure 7:**
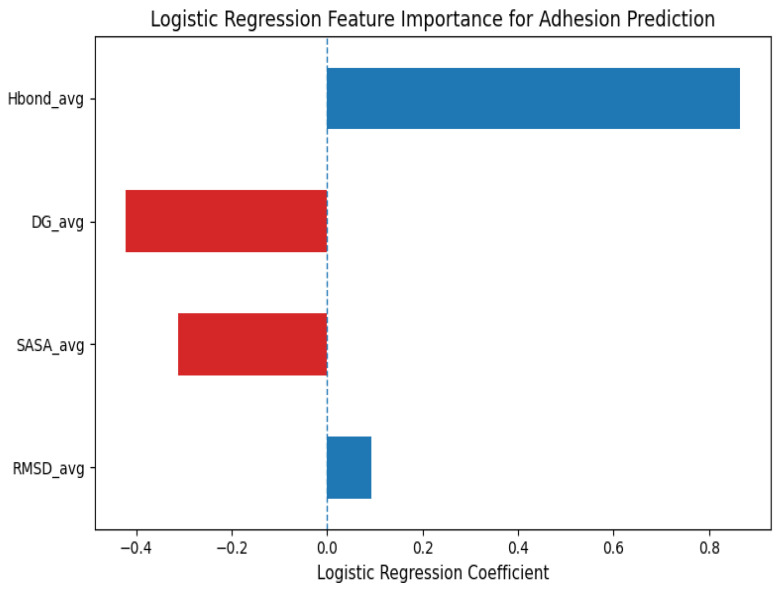
Feature importance from logistic regression-based adhesion prediction. Model coefficients indicate the relative contribution of molecular descriptors to phage–hydrogel adhesion, with positive and negative values representing enhancing and inhibitory effects respectively. Hydrogen bond frequency (Hbond_avg) was the most influential feature, underscoring the key role of hydrogen bonding in stabilizing phage–hydrogel interactions.

To understand the contribution of various molecular descriptors to adhesion prediction, feature importance was calculated using the logistic regression model. As shown in Figure 7, the average number of hydrogen bonds emerged as the dominant positive contributor, whereas ΔG_avg and SASA_avg exhibited negative coefficients, indicating an inverse relationship with the predicted adhesion label.

The trained model resulted in an overall classification accuracy of 50%, reflecting a moderate but non-random level of discrimination between high- and low-adhesion complexes. This level of performance is largely due to the small size of the dataset and the biological complexity of phage–hydrogel adhesion. Furthermore, the efficiency of adhesion is a continuous physicochemical parameter; hence, binary classification necessarily involves borderline instances, especially for complexes with similar structural and energetic characteristics. Consequently, some degree of misclassification is to be expected and should be viewed as a reflection of biological complexity rather than a shortcoming of the ML strategy itself.

Model coefficient analysis yielded mechanistic information regarding the molecular basis of adhesion efficiency. The average number of intermolecular hydrogen bonds had the largest positive coefficient value (0.865), emphasizing the central importance of hydrogen bonding networks in the stabilization of phage–hydrogel interactions. Binding free energy (ΔG) and SASA also had positive coefficients, reflecting the importance of energetic favorability and interfacial contact area in complex formation. By contrast, the average RMSD had a relatively low coefficient value (0.093), suggesting that global conformational stability is of lesser importance than localized interfacial interactions.

In summary, these results clearly show that even with a small dataset, ML models trained on MD-derived descriptors can reproduce the essential physicochemical properties that determine phage–hydrogel adhesion. Although the current model is designed for relative in silico screening rather than direct experimental prediction, it is a compelling proof-of-concept for the integration of machine learning and molecular simulations to uncover the key features of adhesion efficiency.

### 3.6. Diffusion and Release Kinetics of Bacteriophages from Hydrogel Matrices

To assess the applicability of a diffusion model based on Higuchi diffusion principles for the diffusion and release characteristics of bacteriophages in different hydrogel matrices, the adhesion strength calculated using molecular docking, molecular dynamics simulation, and machine learning classification of adhesion strength was used to comparatively estimate the effective diffusion behavior. The release of phages was calculated as the normalized fraction of released phages over a period of 24 h.

In the release kinetics graph, every graph corresponds to a particular phage–hydrogel system, as denoted by the color-coded legend. The differences in the shapes of the graphs are due to the variations in the composition of the hydrogel and the structure of the phage surface proteins. The steeper the graph, the faster the diffusion and the greater the fractional release, while the flatter graph corresponds to lower diffusion rates and a greater retention of phages in the hydrogel matrix.

**Figure 8:**
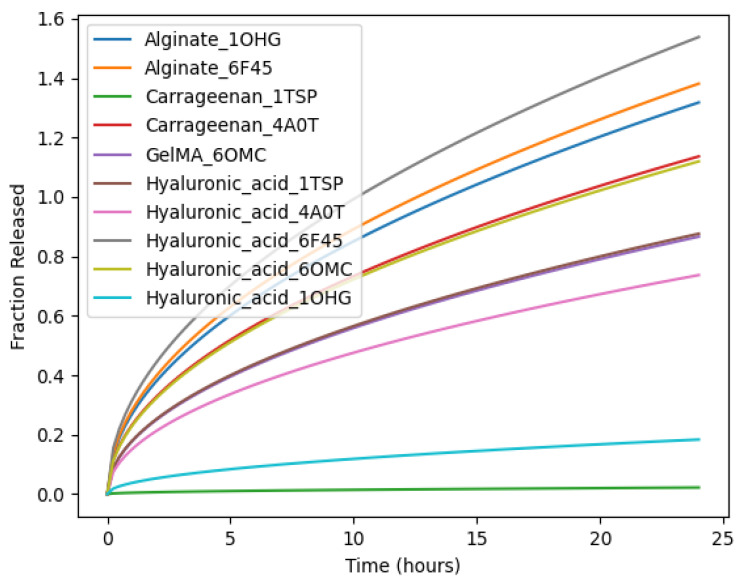
Predicted diffusion and release kinetics of bacteriophages from hydrogel matrices over a simulated 24 h period. Normalized release profiles were estimated using a Higuchi-based diffusion model with effective diffusion coefficients inferred from molecular adhesion strength. Distinct release behaviors reflect differences in phage–hydrogel interaction strength, with stronger adhesion leading to slower release.

As demonstrated in Figure 8, hydrogel systems with better phage–hydrogel adhesion had slower and more sustained release profiles, whereas weaker contacts led to quicker diffusion.

Some of the phage–hydrogel systems, such as Alginate6F45 and HyaluronicAcid6F45, had relatively steeper graphs, indicating fast diffusion rates and greater cumulative releases. This is an indication of weaker intermolecular forces between the phage surface proteins and the hydrogel polymers, resulting in higher molecular mobility and burst release properties.

On the other hand, complexes Carrageenan1TSP and HyaluronicAcid10HG have been found to have a slower rate of release, as indicated by the lower slope values and lower fractional release values over a calculated period of simulation. These patterns are normally associated with higher molecular adhesion values, higher hydrogen occupancies, and lower diffusion rates that are present in sustained phage releases. A middle ground in terms of release rate may be found for complexes such as Alginate10HG and HyaluronicAcid1TSP, which displayed moderate slope without an initial burst phase. Such a pattern of diffusion is consistent with a balance of phage retention and controlled release and would be appropriate for therapy that needs phages to be sustained.

In summary, the predictions of the simulation of the diffusion and release process are consistent with the findings of the molecular docking, MD simulation, and machine learning studies, confirming the validity of the hypothesis that the strength of the molecular adhesion plays an important role in the regulation of the diffusion of phages in the matrix of hydrogel materials. The release profiles of the different phage–hydrogel systems present options in the selection of systems suitable for fast release, controlled release, and sustained release.

## 4. Discussion

Multidrug resistance (MDR) is a big challenge nowadays, and it is predicted that if new methods are not discovered or formulated to treat the deadly pathogens, then there will be no effective antibiotics available until 2050 [41]. Bacteriophage therapy is one of the most promising alternative of available antibiotics for the treatment of multidrug-resistant infections [42]. However, it faces a number of challenges due to the limited in vivo stability, prompt clearance from infectious areas, and vulnerability to environmental inactivation [4,43]. Hydrogel-based delivery systems have shown very effective biocompatibility, flexible physicochemical characteristics and ability to carry biologically active agents [44,45,46]. Although phage-loaded hydrogels are getting a lot of attention, the specific molecular mechanism that decides how phages actually bind with the polymers has received little attention.

Computational methods, including machine learning and artificial intelligence, are increasingly used for the identification and screening of novel drug targets [47]. As we all know, large-scale omics data is available in the context of disease; therefore, machine learning algorithms are very helpful in facilitating the prioritization of potential therapeutic targets with better accuracy and efficiency [48]. Furthermore, advanced computational techniques such as molecular dynamics (MD) simulations, computational immune modeling (C-Imm), molecular docking, and network-based approaches provide deeper insights into protein–ligand interactions, structural stability, immune responses, and system-level effects [49]. Together, these in silico methods proved to be very effective and accelerated the drug discovery pipeline, hence reducing experimental cost and time. In addition, network pharmacology and reverse vaccinology significantly improve target identification and therapeutic design by enabling drug and gene interactions and rational multi-epitope-vaccine (MEV) development [50,51,52].

This study offers a unified computational approach to better understand phage–polymer interactions at the molecular level. We worked on molecular-scale adhesion mechanisms and macroscopic release kinetics by using molecular docking, MD simulations, machine learning-driven classification and diffusion modeling. Molecular docking analyses of hyaluronic acid, alginate, carrageenan, and chitosan showed stronger binding affinities toward phage structural proteins compared to neutral synthetic polymers such as polyethylene glycol (PEG) and polyvinyl alcohol (PVA). These hydrogel polymers contain charged and polar functional groups that offer high water absorption capacity and strong hydrophilicity [53,54]. Notably, docking and simulation analyses revealed that glycosaminoglycan-based polymers presented stable interactions with structurally distinct phage proteins. These interactions also reflect conformational flexibility and multivalent interaction capability. Previous investigations of virus–polymer interactions found that polymer composition, functional groups and intermolecular pressures influence viral adherence at material interfaces [55,56].

The observed discrepancies in contact strength between bacteriophage proteins and the examined hydrogel systems can be attributed to variances in their physicochemical properties and molecular structures. Hydrogels differ in functional groups, charge distribution, hydrophilicity and polymer backbone flexibility, all of which have a substantial impact on how they interact with biological macromolecules. Hydrogels with a high concentration of hydroxyl, carboxyl or amino functional groups may generate stronger hydrogen bonds and electrostatic interactions with bacteriophage protein surface residues. In contrast, more neutral polymers, such as polyethylene glycol, interact predominantly via weaker van der Waals and hydrophobic interactions. Furthermore, polymer chain flexibility and cross-linking density might influence the accessibility of interaction sites and the stability of phage–hydrogel complexes. These chemical properties all contribute to observed variances in docking scores, interaction patterns and anticipated adhesion efficiency among hydrogel systems.

Binding site prediction revealed that most polymer interactions were found to be localized in surface-exposed residues rather than in internal structural domains. Although these findings suggest that the global capsid conformation is likely conserved and receptor-binding regions may remain accessible, the direct impact of polymer interactions on phage infectivity cannot be identified without experimental validation. Strong polymer adherence may interfere with receptor-binding regions if interactions occur near tail fibers or host recognition domains. However, docking and MD investigations revealed interactions only with surface-exposed residues, with no large structural changes, implying that polymer binding is likely reversible, but experimental infectivity assays are necessary for confirmation [57]. Significantly, short polymer fragments were used in our docking analysis; thus, multivalent binding effects in a three-dimensional hydrogel matrix could be quantitatively different [58]. To mitigate computational limitations, monomeric units were employed as representative segments to encapsulate the principal interactions between hydrogel functional groups and the phage protein surface. Real hydrogels generate cross-linked polymer networks with multivalent binding and structural flexibility, whereas the monomer-based approach identifies probable binding sites and critical interaction residues. In actual hydrogel matrices, numerous polymer chains may interact with the protein surface at the same time, thereby enhancing adhesion via cooperative effects [59].

MD simulations also supported the dynamic stability of all the docked complexes. Molecular dynamics simulations, including analyses of RMSD, RMSF, R_g_ and SASA, showed that the protein remained structurally stable and did not undergo global unfolding upon polymer binding. The predominance of non-covalent interactions further suggests reversible adhesion, which is an ideal application for controlled release systems [60,61]. While molecular docking employed a mainly stiff receptor approximation, later MD simulation allowed for limited conformational flexibility of the protein structures during the simulation timescale. This dynamics analysis partially captures structural variations in surface-exposed areas such as receptor-binding domains. However, large-scale conformational rearrangements that may occur during actual host recognition events were not explicitly addressed and they remain a key area for further research. However, computer simulations are only limited to the nanosecond timescale; consequently, these simulations might have missed slower conformational rearrangements that can effect long-term behavior [62,63]. Although the 100 ns molecular dynamics simulation revealed significant information about the stability and interaction dynamics of the phage–hydrogel complexes, longer simulations may be necessary to capture slower conformational changes such as protein structural rearrangements or polymer relaxation. Future research, including longer simulation timeframes and multi-scale modeling, could provide more insight into the long-term behavior of phage–hydrogel systems in physiological contexts.

The machine learning-based adhesion classification model provided additional mechanistic insight, identifying hydrogen bond occupancy as the most influential predictive feature. Although the dataset was limited and derived from simulation outputs, the model demonstrates the feasibility of integrating molecular descriptors into predictive screening pipelines for hydrogel selection. Expansion with experimentally validated datasets would enhance generalizability and reduce overfitting risk [64].

Importantly, diffusion modeling established a mechanistic link between molecular adhesion strength and macroscopic release kinetics. Stronger predicted phage–polymer interactions corresponded to reduced diffusion coefficients and sustained-release profiles, whereas weaker interactions resulted in rapid diffusion and burst release behavior. While the model assumed homogeneous matrix properties and Fickian diffusion, the observed trends provide a rational framework for tailoring hydrogel chemistry to specific therapeutic contexts [65]. Interestingly, comparable interaction patterns were observed across multiple phage structural proteins, suggesting that polymer chemistry may exert a dominant influence under the modeled conditions. Nevertheless, phage-specific surface architecture, tail fiber flexibility, and host recognition domains may modulate these interactions in biological environments [66].

Several limitations warrant consideration. Polymer models were simplified and did not incorporate cross-link density, mesh size heterogeneity, or entanglement effects characteristic of real hydrogels. Biological variables such as enzymatic degradation, immune clearance, biofilm penetration, variations in pH, ionic strength and the complex composition of biological fluids were not included, all of which may influence polymer–phage interactions under physiological conditions. Furthermore, infectivity preservation was inferred from structural stability rather than experimentally measured plaque-forming ability [67]. Although the current study provides computational insights into phage–hydrogel interactions, experimental validation utilizing techniques such as spectroscopic characterization or in vitro phage release assays might help to reinforce the findings. Future research may include experimental efforts to validate the proposed interaction mechanisms. Future work integrating coarse-grained polymer simulations, longer-timescale MD, and experimental validation will be essential to translate these computational findings into clinically relevant systems. Overall, this study establishes a mechanistic and predictive computational platform for rational hydrogel design in bacteriophage delivery applications. By linking molecular adhesion determinants with diffusion behavior, it provides a foundation for optimizing controlled-release phage therapeutics in the context of antimicrobial resistance.

## 5. Conclusions

This study provides a computational approach for rationalizing hydrogel-based bacteriophage delivery systems, considering molecular interactions in relation to release characteristics. The strategy used molecular docking, MD simulations, ML, and diffusion modeling in providing mechanistic insights into the influence of hydrogel chemistry on phage protein adhesion, structural integrity, and mobility. Hydrogels containing more polar and charged functional groups, such as hyaluronic acid, alginate, carrageenan, and chitosan, facilitated more significant and stable yet reversible protein phage adhesion, supported by hydrogen bonding and electrostatic mechanisms. Importantly, dynamic simulations proved that protein–polymer interaction does not influence the structural integrity of phages, justifying the possibility of using hydrogel in encapsulating bacteriophages. The presence of specific interaction patterns for various proteins in the phage system signifies that the polymer composition has a more significant role to play compared to the proteins. Thus, the results signify that the optimized hydrogel formulation can be widely used for various phage systems. By establishing the relationship between the adhesion strength and diffusion characteristics, predictions can be made regarding the release kinetics that are specific to the requirements. This cost-efficient approach makes the computational method all the more significant in the design of the delivery system. Thus, in conclusion, the research results highlight the significance of the rational design of hydrogel together with the phage system, and the significance of the ongoing trend of using the concept of phages for overcoming antimicrobial resistance.

## Figures and Tables

**Table 1:** Physiochemical properties of selected hydrogel polymers.

Hydrogel	Monomer/Building Unit	PubChem ID	Molecular Weight (g/mol)	Functional Groups	Charge Nature	Biomedical Relevance
Chitosan	D-Glucosamine	439213	179.17	-NH_2_, -OH	Positively charged	Antimicrobial delivery, wound healing
Alginate	Sodium alginate	5102882	216.12	-COOH, -OH	Negatively charged	Encapsulation-controlled release
Hyaluronic acid (HA)	Repeating disaccharide unit	155618327	425.38	-COOH, -OH	Negatively charged	Tissue regeneration, drug delivery
Carrageenan	3,6-Anhydro-D-galactose	16069996	162.14	-OH	Neutral	Structural gel component
Carrageenan	D-Galactose-4-sulfate	22826301	260.22	-SO_3_^−^, -OH	Negatively charged	Gel formation, sustained release
Polyethylene glycol (PEG)	Ethylene glycol	174	62.07	-OH	Neutral	Biocompatible coatings, drug delivery
Polyvinyl Alcohol (PVA)	Vinyl Alcohol (ethanol)	3082248	155.19	-OH	Neutral	Tissue engineering, hydrogel matrices
GelMA	N-methacryoyl-L-lysine	18634837	214.26	-NH_2_, -COOH, -C=C-	Amphoteric	Injectable hydrogels, biofabrication
HEMA	2-Hydroxyethyl methacrylate	13360	130.14	-OH, -C=C-	Neutral	Cross-linked biomedical hydrogel

**Table 2:** Docking scores and interaction parameters of hydrogel polymers with bacteriophage proteins.

Protein Phage	Hydrogel	Binding Affinity (kcal/mol)	RMSD (Å)	Interacting Residues
Mature Capsid Protein (1OHG)	Hyaluronic acid	−5.5	1.26	GLU A: 234, ASN A: 182, GLN A: 179, GLY A: 230, LEU A: 368, THR A: 180
	Alginate	−5.1	0.69	ASN A: 182, ASN A: 232, GLY A: 230, GLU A: 234
	Chitosan	−4.9	0.54	GLY A: 228, ASN A: 232, ASN A: 182, LEU A: 368
	Carrageenan	−4.8	1.64	GLY A: 230, LYS A: 184, ASN A: 182, GLY A: 228, GLU A: 234
	GelMA	−4.6	0.14	ASN A: 182, LYS A: 184, ASN A: 232, ALA A: 181
	HEMA	−3.7	0.44	ASN A: 182, LEU A: 233, ALA A: 181
	PVA	−3.6	0.29	ASN A: 182, LEU A: 233, ALA A: 181
	PEG	−2.8	0	ASN A: 182, LEU A: 368
P22 Tailspike Protein (1TSP)	Hyaluronic acid	−5.7	0.49	HIS A: 565, LYS A: 566, ASN A: 536, VAL A: 537. ALA A: 538, LYS A: 569
	Alginate	−4.7	1.06	VAL A: 537, ILE A: 535, HIS A: 565
	Carrageenan	−4.7	1.96	VAL A: 537, ASN A: 539, LEU A: 540
	Chitosan	−4.3	0.49	SER A: 578, ASP A: 556, TYR A: 555, SER A: 552
	GelMA	−4.3	1.25	ARG A: 563, VAL A: 537, ILE A: 535, LEU A: 540, HIS A: 565
	HEMA	−3.5	0.57	VAL A: 537, ILE A: 535, HIS A: 565, LEU A: 540
	PVA	−3.2	0.37	LYS A: 631, ASP A: 632, ILE A: 630, LEU A: 637
	PEG	−2.6	0.22	THR A: 515, GLN A: 489
T7 Tail Fiber gp17 (4A0T)	Hyaluronic acid	−5.7	1.70	ASP A: 461, ARG A: 460, PHE A: 463, GLU A: 553, ARG A: 508
	Carrageenan	−4.8	0.83	ARG A: 460, SER A: 462, ARG A: 508, PHE A: 463, ALA A: 465, PHE A: 492
	Alginate	−4.7	0.45	ALA A: 465, ARG A: 508, ASN A: 494
	Chitosan	−4.5	0.99	PHE A: 492, GLUA A: 553, ALA A: 465
	GelMA	−4.4	1.63	ASN A: 552, GLU A: 551, ALA A: 465, ARG A: 460, VAL A: 464
	PVA	−3.9	0.54	ALA 465, VAL A: 464
	HEMA	−3.4	0.39	ASN A: 494, ARG A: 508, VAL A: 464
	PEG	−2.4	0.09	ARG A: 493, ARG A: 491
S16 Long Tail Fiber Protein (6F45)	Hyaluronic acid	−4.7	0.61	GLY A: 612, HIS A: 614, THR A: 609, ILE A: 615
	Alginate	−3.7	0.48	GLU A: 610
	Carrageenan	−3.7	1.13	THR A: 609, GLY A: 612, LYS A: 613
	Chitosan	−3.4	0.31	LYS A: 613, GLY A: 612, HIS A: 614, THR A: 609
	GelMA	−3.3	1.15	THR A: 609, LYS A: 613, ILE A: 615, HIS A: 614
	PVA	−2.9	0.61	ASN A:603, PHE A: 608
	HEMA	−2.8	0.95	ASN A: 603, PHE A: 608
	PEG	−2	0.02	LYS A: 613, THR A: 609
Capsid Protein gp10 (6OMC)	GelMA	−5.6	1.75	TYR A: 383, LEU A: 344, LEU A: 385, LEU A: 346, LEU A: 330, LEU A: 341, ILE A: 327, VAL A: 404
	Hyaluronic acid	−4.7	1.62	ARG A: 336, ARG A: 332, LYS A: 333, LEU A: 334, GLY A: 335, ASP A: 306
	Alginate	−4.6	0.45	GLY A: 338, LEU A: 334, ARG A: 331, GLY A: 335, GLY A: 449
	Chitosan	−4.6	0.13	ASN A: 448, VAL A: 450, ALA A: 447, ASP A:306
	PVA	−4.3	0.48	LEU A: 334, LEU A: 330, LEU A: 344, ARG A: 331, VAL A: 404, LEU A: 346
	Carrageenan	−4.2	0.71	LYS A: 333, SER A: 307, ASP A: 306, VAL A: 450, GLY A: 449, ALA A: 447
	HEMA	−4.1	0.84	ARG A: 331, ILE A: 327, LEU A: 285, LEU A: 346, LEU A: 341, LEU A: 344
	PEG	−2.9	0.42	GLY A: 449, ASP A:306

**Table 3:** MM/PBSA binding free energy components for selected phage–hydrogel polymer complexes obtained from 100 ns molecular dynamics simulations.

Complexes	ΔG_bind (kcal/mol)	ΔG_vdw	ΔG_Hbond	ΔG_polar
1OHG-Hyaluronic acid	−41.65	−27.13	−2.68	36.63
1OHG-Alginate	−30.69	−20.86	−1.40	18.59
1TSP-Hyaluronic acid	−44.51	−24.85	−1.61	27.74
1TSP-Carrageenan	−47.29	−21.53	−1.44	27.59
4A0T- Hyaluronic acid	−52.32	−38.91	−0.77	23.71
4A0T-Carrageenan	−35.39	−31.77	−3.06	29.76
6F45-Hyaluronic acid	−15.37	−20.97	−0.96	20.34
6F45-Alginate	−27.94	−8.46	−1.14	11.13
6OMC-GelMA	−61.58	−30.83	−0.24	13.20
6OMC-Hyaluronic acid	−39.37	−28.95	−0.50	14.82

**Table 4:** Integrated molecular dynamics and machine learning-derived interaction metrics for phage–hydrogel polymer complexes.

Complex	Avg_RMSD (Å)	Avg_Hbond	Avg_ΔG (kcal/mol)	Avg_SASA (nm^2^)	Adhesion_Label
Hyaluronic_acid_1OHG	12.78	12.29	−250.96	374.33	1
Alginate_1OHG	8.17	12.18	−22.14	285.13	0
Hyaluronic_acid_1TSP	8.30	15.49	−36.55	387.34	1
Carrageenan_1TSP	7.32	18.46	−2214.98	126.87	1
Hyaluronic_acid_4A0T	18.24	25.16	−38.34	297.66	1
Carrageenan_4A0T	19.42	10.96	−28.68	346.81	0
Hyaluronic_acid_6F45	7.31	13.55	−14.92	337.98	0
Alginate_6F45	7.08	9.46	−23.83	217.68	0
GelMA_6OMC	9.53	10.78	−44.34	143.22	1
Hyaluronic_acid_6OMC	10.51	8.01	−31.21	453.89	0

## Data Availability

The data presented in this study are available within the article. No additional datasets were generated or analyzed during the current study.

## Abbreviations

The following abbreviations are used in this manuscript:

**Abbreviation**	**Definition**
Å	Angstrom
AI	Artificial Intelligence
AMBER	Assisted Model Building with Energy Refinement
CASTp	Computed Atlas of Surface Topography of Proteins
CASTpFOLD	CASTp integrated with AlphaFold-based structures
C-Imm	Computational Immune Modeling
Cl^−^	Chloride Ion
ΔG	Binding Free Energy
ΔG_bind	Binding Free Energy of Binding
D_eff	Effective Diffusion Coefficient
ff14SB	AMBER Force Field 14SB
GelMA	Gelatin Methacryloyl
HEMA	2-Hydroxyethyl Methacrylate
HPMC	Hydroxypropyl Methylcellulose
k_H	Higuchi Release Constant
LOOCV	Leave-One-Out Cross-Validation
MDR	Multidrug-Resistant
MD	Molecular Dynamics
MEV	Multi-Epitope Vaccine
ML	Machine Learning
MM/PBSA	Molecular Mechanics/Poisson–Boltzmann Surface Area
MM/GBSA	Molecular Mechanics/Generalized Born Surface Area
Na^+^	Sodium Ion
NPT	Constant Number of Particles, Pressure, and Temperature Ensemble
OPLS	Optimized Potentials for Liquid Simulations
PEG	Polyethylene Glycol
PDB	Protein Data Bank
PVA	Polyvinyl Alcohol
PyRx	Python Prescription Virtual Screening Tool
Q	Cumulative Amount of Drug Released
Rg	Radius of Gyration
RMSD	Root Mean Square Deviation
RMSD_avg	Average Root Mean Square Deviation
RMSF	Root Mean Square Fluctuation
SASA	Solvent Accessible Surface Area
SASA_avg	Average Solvent-Accessible Surface Area
SDF	Structure Data File
TIP3P	Transferable Intermolecular Potential 3-Point Water Model
